# Apply the SERVQUAL Instrument to Measure Service Quality for the Adaptation of ICT Technologies: A Case Study of Nursing Homes in Taiwan

**DOI:** 10.3390/healthcare8020108

**Published:** 2020-04-24

**Authors:** Chao-Hua Ko, Chin-Mei Chou

**Affiliations:** Department of Industrial Engineering and Management, Yuan Ze University, Chung-Li 32003, Taiwan; ke.stacy@gmail.com

**Keywords:** e-Health, service quality, SERVQUAL model, nursing homes

## Abstract

The adoption of information and communication technology by elderly care organizations is an inevitable trend. Most empirical studies on e-Health service quality have focused predominantly on the general population rather than on the elderly. Thus, the generalizations are rather problematic. In addition, in the planning stage, pre-implementation analysis is considered critical but seldom performed. In this research, an instrument to evaluate the e-Health service quality in nursing homes was developed based on the SERVQUAL model. Furthermore, a pre-implementation analysis combining the SERVQUAL questionnaire and importance performance analysis was performed. Dissatisfactory factors were identified as follows. Regarding the physical environment quality, the residents expressed that the nursing homes did not provide well-maintained rooms and that the temperature in the rooms was unsuitable. Regarding the outcome quality, the elderly residents replied that the medical treatments and doctor visits were not well scheduled. Regarding the interaction quality, the residents indicated that the staff did not solve their problems sincerely or clearly understand their needs. Health care informatics (HCI) such as an electronic shift system (ESS) and electronic health records (EHR) are proposed to eliminate these problems. Given current resource limitations, our instrument and methodology proposed in this research could be extremely meaningful in practical application.

## 1. Introduction

Despite being a widely used term, e-Health has no single universally accepted definition. According to the WHO, e-Health is the transfer of health resources and health care by electronic means [1]. Another definition of e-Health states that it is “the use of electronic devices capable of creating, storing, retrieving, and transmitting of data between end users for the purpose of improving patient safety and quality of care” [2]. An e-Health system consists of not only information and communication technology (ICT) but also socio-organizational and environmental factors and processes. The increased use of ICT in medical practice has been demonstrated to offer the potential for large benefits for health care, such as improved quality of care, patient empowerment, cost savings, and the promotion of behavioral change in patients [3,4,5]. The U.S. Government Accounting Office (U.S. GAO) studied the benefits of e-Health in eleven public and private health care delivery organizations of varying sizes and settings that had invested significantly in e-Health. The main benefits were as follows: (1) 50–80% reduction in medication error rates; (2) more than 15% reduction in diagnostic imaging tests because of online access to results; (3) significant reduction in time to refer patients, using online scheduling and communication tools; (4) 40% increase in patient screening and preventative health care procedures; (5) 40% increase in the use of standard protocols by physicians [6].

The adoption of ICT by these elderly care organizations is an inevitable trend and may provide solutions to important problems, but these potential solutions face obstacles. For example, from the perspectives of the information systems success model (ISS model) and the clinical adoption framework (CA Framework), system quality, information quality, and service quality are considered the three critical constructs of the successful adoption of ICT for medical organizations [7]. The value of service quality has been recognized by scholars and organizers in recent years. But most empirical studies on service quality have focused predominantly on the general population rather than the elderly. The generalizations of such findings to the elderly population are rather problematic [8]. For instance, the attitudes, motivation, and expectations of using technology products in the general population and the elderly population vary. The elderly often pose resistance to and feel anxiety about these new technologies. These phenomena are worsening because modern technologies have become increasingly complex and the elderly’s mental models might be not supported.

Despite the growing need to evaluate service quality in an e-Health environment, just a few evaluation instruments have been developed to date. This lack is another deficiency in the adoption of ICT by medical organizations. We believe it is necessary to develop an instrument to evaluate the service quality and guide the implementation of ICT in healthcare settings. To summarize, two main contributions are addressed in this research. First, a validated and reliable SERVQUAL (SERVice QUALity) questionnaire specific to nursing homes has been developed to measure the residents’ satisfaction. This questionnaire can reveal multidimensional service defects, and is especially suitable for the pre-adoption evaluation of novel technology for nursing homes. Second, importance performance analysis (IPA) was applied to compensate for the deficiencies of the SERVQUAL in presenting the relationship between performance and importance to draw up recommendations for managers to adapt ICT technologies.

## 2. Literature Review

### 2.1. The Architecture and Applications of e-Health

**Figure 1:**
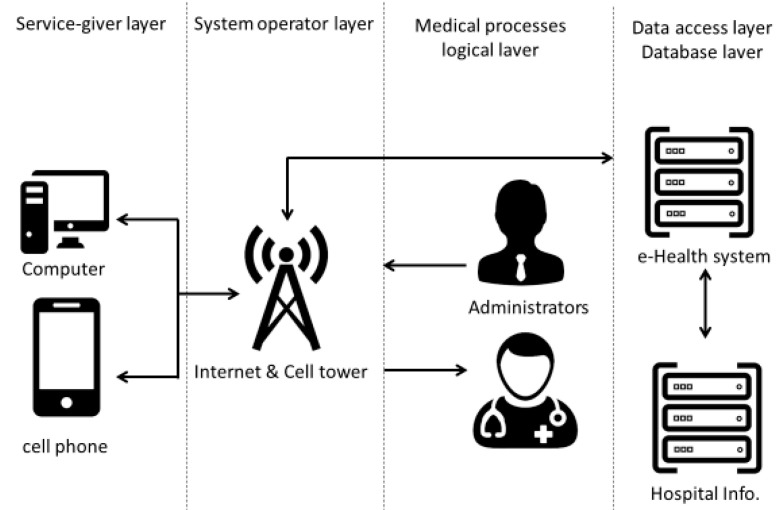
The schematic diagram of e-Health [9].

e-Health systems rely on a distributed service infrastructure and the underlying end-to-end communication system for service delivery. This system is provided by multiple network communication (shown as Figure 1) [9]. e-Health can deliver different services to users and medical staff, including telemedicine services and monitoring services, sharing both related information and available resources. The e-Health service domain varies with respect to application area, application purpose, content type, and context of use. In general, the architecture of e-Health contains a service-provider layer, a communication layer, a medical processes layer, and a database layer [9]. Hamilton proposed a set of ICT functions for the specific needs of nursing home environments, included electronic supportive documentation, point-of-care, assessment and care planning, electronic prescribing, computerized physician order entry (CPOE), medication administration records (MAR), and electronic health records (EHR) [10].

### 2.2. The Evaluation Framework for ICT Adoption

Depending on the content and context of the stages being evaluated, different frameworks and models can be applied. In the design stage, managers may evaluate whether the specifications of the system meet its function. The information system success model (ISS model) and clinical adoption framework (CA Framework) are two of the most widely used models and frameworks that describe the evaluations of information systems as a multidimensional construct.

**Figure 2:**
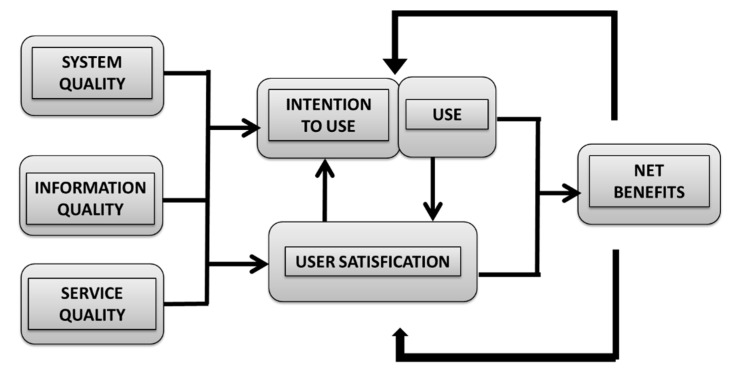
Updated information system success model [7].

The original ISS model was derived from a review of 180 conceptual and empirical information system studies in different fields by DeLone and McLean [11]. After 10 years, they updated the ISS model based on empirical findings from 285 peer-reviewed papers published in academic journals between 1992 and 2002. In the updated model, a service quality dimension was added, and the individual and organizational impact dimensions were combined as a single construct, named net benefits. The four dimensions of the updated ISS model are system quality, information quality, service quality, and net benefits (shown as Figure 2) [7]. In this updated model, service quality refers to staff reliability, empathy, and responsiveness. Each of these dimensions successfully represents a distinct construct of adoption. The updated ISS model is one of the few models that have been empirically validated in numerous laboratory and field studies across educational, business, and healthcare settings.

**Figure 3:**
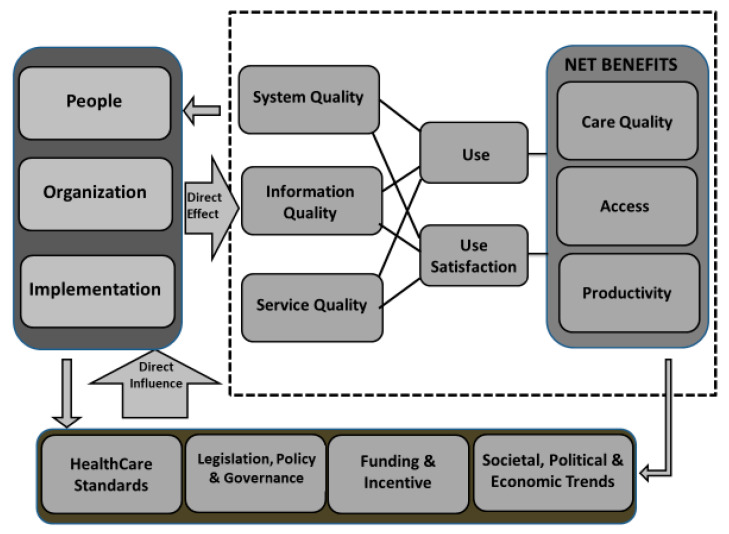
The schematic diagram of clinical adoption framework [12].

Based on theories and models from information systems, organization science, and health informatics, the CA Framework allows micro-, meso-, and macro-level views of how e-Health is adopted by medical staff in different settings (shown in Figure 3) [12]. The first level, the micro-level, focuses on the quality of the components; that is, the information, system, and service of e-Health systems. The second level, the meso-level, focuses on the dimensions of people, organization, and implementation, all of which directly affect the micro-level. The final level, the macro-level, focuses on environmental factors that directly influence the meso-level. These factors are the social trends, funding, standards, and healthcare governance. Each level has a feedback loop that allows the results of ongoing efforts to adopt the system to affect the higher levels [12]. In this framework, service quality means the responsiveness during the implementation of the e-Health system, the training, and ongoing support by medical staff, but the service empathy and assurance of the ISS model are not included. This CA framework serves as an overall model of e-Health adoption. To achieve success with an e-Health system, it is important that the micro-, meso-, and macro-level factors of this framework all be addressed. It is worth noting that, our instrument developed in this research can firm up the measurement of service quality in nursing homes. According to CA framework, service quality has impact on micro-level, user satisfaction, and net benefits in terms of care quality, productivity, and access.

### 2.3. The Evaluation of Service Quality in Nursing Homes

Service cannot be displayed, demonstrated, standardized and it needs a high degree of customer involvement in the delivery process. Nevertheless, there is a general perspective that service quality is a multidimensional construct. Based on the characteristics, it is hard for service organizations to evaluate and provide a stable service quality to customers. Thus, the development of instruments for evaluating the quality of service is a crucial step and should be highlighted by employing multiple-dimension indicators [13]. In general, service quality measurement can be carried out in three different approaches: (1) Surveys and questionnaires. The most common kind of customer insight survey for exploring service quality is the questionnaire survey. Questionnaires are inexpensive, are comparable with other research, cover numerous aspects of a topic, and offer actionable information. (2) Post service investigation. This is the practice of asking customers to rate the service right after service is been delivered. Different scales can be used for the post service rating. Many make use of a number rating from 1 to 10. (3) Objective service metrics. For instance, first response time shows how quickly a customer receives a response on her inquiry and the average queueing waiting time presents the time customers have to wait to be served. This quantitative analysis may be not enough to judge the quality of service directly, but they play a crucial role in showing the areas should be improved.

To evaluate the service quality in nursing homes is a complicated task because of the elderly subjects and complex nature of health care. Questionnaires are considered as an appropriate method. This approach is especially suitable for evaluating feelings, beliefs, and attitudes, and it is often used to measure service quality in different fields. The questionnaire survey is used by many researchers for evaluating the feelings about and attitudes toward e-Health systems applied in medical institutions [13]. Furthermore, questionnaires are inexpensive, are comparable with other research, cover numerous aspects of a topic, and offer actionable information.

To increase healthcare service quality, Taipei Medical University Hospital has implemented an e-Health system. Its primary function is to help medical staff and hospital administrators manage individual patients in a systematic fashion [9]. Questionnaires were distributed before and after the implementation of the e-Health system. The results showed that patients who adopted the e-Health system were satisfied with its functions and were willing to continue using this advanced system for illness treatment, illness prevention, and patient service dimensions. Chao and his colleague concluded that the e-Health system not only improved the quality of the healthcare service but also improved patient relationships with healthcare providers [9]. Otieno and his colleagues reported that although the use of e-Health systems in hospitals is steadily increasing, no validated instruments had been used to assess the effectiveness of these systems from the viewpoint of nurses [14]. They developed an instrument to measure nurses’ views on the use, quality, and satisfaction dimensions of e-Health systems. Their results showed that the constructs of the use and quality dimensions were positively correlated with user satisfaction. Thus, a reliable and valid questionnaire with 34 items has been developed for use in evaluating e-Health systems in hospitals from nurses’ perspectives [14].

To identify existing deficiencies in e-Health systems, an evaluation framework for hospitals utilizing such systems was developed by Stylianides, Antonis et al. [15]. Their framework was constructed for three main areas, identified as human factor, technology, and organization. They claimed that their instrument could provide a holistic image of e-Health by evaluating any hospital system [15]. Sabur Safi and his colleague [16] analyzed the acceptance of new health care technologies by applying the technology acceptance model to data obtained in Germany. Their survey used questionnaires to gain insight into digital health applications in a sample of 9621 patients with acute and chronic conditions and in healthy users. Significant differences were observed among the age groups and genders of the respondents. For example, relatively lower acceptance rates were observed in older individuals; also, men were more likely to accept digital technologies, while women preferred coaching and consultation apps [16].

### 2.4. The Service Quality Questionnaires

To evaluate the acceptance of e-health in nursing homes is a complicated task because of the complex nature of health care and the high sensitivity of acceptance level to socio-cultural variations. According to Akter et al. [17], subjective satisfaction is a dynamic, multidimensional, and comprehensive indicator which can be used to evaluate the interactions between humans and systems efficiently. This viewpoint was confirmed by subsequent empirical research [14]. In current service literature, the SERVQUAL model [18,19,20] and health service quality scale [21] have been the two major model used to measure service quality in healthcare settings. The unique attributes of service have made it difficult to apply knowledge of physical quality measurement to the service domain. First, because of the intangibility of service, it cannot be displayed, demonstrated, or illustrated concretely. Second, service cannot be standardized. The performance of services is dependent to some extent on the level of demand. Third, there is a high degree of customer involvement in the delivery of service. Parasuraman et al. [18,19,20] proposed the customer’s perception of service quality based on a gap model, identifying five major gaps in the service quality concept. The five major gaps are the knowledge gap, the standards gap, the communications gap, the delivery gap, and the expected and service quality gap. Service quality as defined by the gap model can be examined by comparing the customers’ expectations with their perceptions of the performance of the service provider. Under this concept, the SERVQUAL questionnaire was developed by Parasuraman and his colleagues [18,19,20].

(1) Gap1: The knowledge gap. Differences exist between the market’s expected service and management’s perceptions of the market’s expected service.
(2) Gap2: The standards gap. Differences exist between management’s perceptions of customers’ expectations and the translation into service procedures and specifications.
(3) Gap3: The delivery gap. Differences exist between service quality specifications and the service actually delivered.
(4) Gap4: The communications gap. Differences exist between service delivery intentions and what is communicated to the customer.
(5) Gap5: The expected and perceived service gap. Differences exist between the customers’ expectations and their perception of the actual service delivered.

This self-report questionnaire contains twenty-two items covering five dimensions (reliability, assurance, tangibles, empathy, and responsiveness). The items are scored on a seven-point Likert-type scale. The SERVQUAL is used to diagnose the strengths and weaknesses of service quality, and it is widely regarded as the most comprehensive instrument for this assessment.

However, apart from its wide use, several theoretical and operational criticisms of the measurement model have been pointed out [22]. In theoretical aspects, there is little evidence that customers access service quality in terms of perception (P) minus expectation (E) gaps. Furthermore, SERVQUAL focus on the process of service delivery, not on the outcomes of the service encounter, while process and outcome together are a better predictor than process or outcome alone. Finally, SERVQUAL five dimensions are not universal. In other words, items do not always load on to the five dimensions proposed by Parasuraman and his colleagues. In operational aspects, the term expectation is polysemic and consumers use different concepts other than expectations to evaluate service quality. In addition, the seven -point Likert scale has been criticized on several grounds, for instance, it has been criticized for its lack of verbal labeling for points two to six. This will cause respondents to overuse the extreme ends of the scale. Finally, two administration of the instrument always causes repetitiveness and confusion. Respondents appear to be confused by the two administration of the expectation and the perception versions of the SERVQUAL, which will result in imperil data quality. 

**Figure 4:**
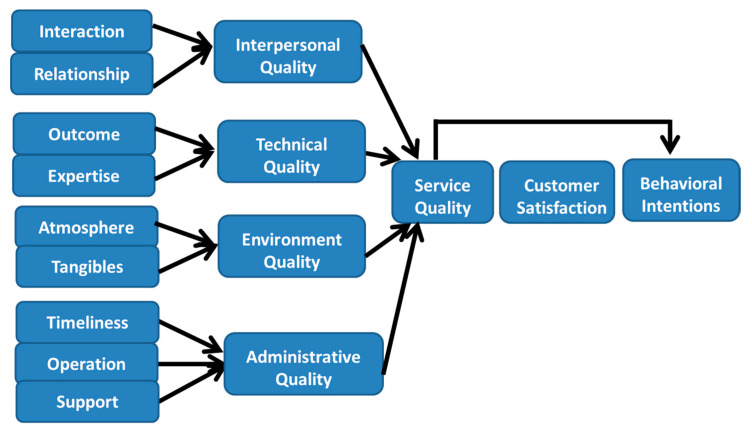
The concept of hierarchical model of health service quality [22].

A multidimensional hierarchical scale for measuring health service quality is developed and validated by Dagger and his colleague in 2007 [21]. Their model identified nine subdimensions driving four primary dimensions, which in turn were found to drive service quality perceptions. The primary dimensions were interpersonal quality, technical quality, environment quality, and administrative quality. The subdimensions were interaction, relationship, outcome, expertise, atmosphere, tangibles, timeliness, operation, and support (shown as Figure 4). 

Under their hierarchical structure, executors are able to measure service quality at overall level (with a global measure of service quality), at the primary dimension level (with overall measures of four primary dimensions), and at the subdimension level (with measures of nine subdimensions). Executors can measure service quality at any one or all of these levels depending on their information requirements. Furthermore, this model can be used as a diagnostic tool for identifying poor and excellent service performance. The scale can be used to benchmark across multiple functions, across multiple locations, or within a specific industry; in addition, any of these situations can also be compared across time.

## 3. Methodology

### 3.1. Choice and Development of SERVQUAL Questionnaires

According to Opoku, the researcher would determine the decisions of the study design based on the problem definition, the research objectives, and the researcher’s interest as to the methodology that is able to provide meaningful results toward the goals [23]. The theory of how research should be undertaken, including the practical and theoretical considerations. For instance, on account of the time and costs involved, a researcher might be constrained to settle for sub-optimizing research design. Opoku has identified six elements of research design to support researchers to achieve the goals efficiently: (1) Purpose of the study; (2) type of investigation; (3) extent of researcher interference; (4) study setting; (5) unit of analysis; (6) the time horizon [23].

In this research, questionnaire is considered as an appropriate instrument based on the features of service quality and the limitations of elderly participants. Service quality is a multidimensional with hierarchical structure, whose measurement must gather the evaluation of subjective feelings and judgements. In addition, elderly people often have reduced mental and cognitive ability. It takes more time and is difficult to follow complex experimental processes.

The SERVQUAL questionnaire is an easy-to-use, multidimensional, and comprehensive instrument which can be used to evaluate the subjective feelings. It has acceptable general validity, and since its development, numerous service industries, such as finance, communications, higher education, healthcare, and information technology, have employed it [24,25,26,27,28]. Furthermore, it is widely accepted for its conceptualization and assessment of service quality. In a literature review of the SERVQUAL model from 1998 to 2013 by Wang and his colleagues, the SERVQUAL model was found to be a hot research topic of academic researchers and a significant contributor to service quality research [29].

The SERVQUAL instrument presents general quality dimensions for service industries; it does not include specific service attributes for researchers. In this study, we developed and modified our own SERVQUAL questionnaire based on the results of pilot studies and the extant literature on nursing homes. To examine the comprehensibility of the questionnaire, the pilot study included 26 attributes of service. In the end, we determined that 23 of these attributes were consistent with the dimensions of the SERVQUAL. The final version of the questionnaire consisted of five dimensions (Table 1) and 23 attributes (Table 2).

One of the most basic parts of instrument development is to consider its validity and reliability. Validity has been defined and characterized as the degree to which the instrument really does what it implies to do. Reliability means the stability or consistency of questionnaire scores over time or across raters. In this research, the reliability and validity of our SERVQUAL questionnaires is determined by Cronbach’s alpha and Pearson’s correlation coefficient. The standard value of Cronbach’s alpha for reliability is usually 0.7 [30]. For this instrument, the Cronbach’s alpha was 0.87, indicating that our questionnaire was sufficiently reliable. Pearson’s correlation coefficient was used to examine the correlation between the total score and the subject real performance. In general, a coefficient >0.7 indicates strong validity and a higher coefficient indicates a better data fit to the measurement objective the overall coefficient for our questionnaires was sufficiently validated under the general criteria. For nursing homes which are prepared to apply ICT, this questionnaire can provide a comprehensive and practical guide to adopting ICT with minimum disruptions. For nursing homes that have applied ICT technologies, this questionnaire can identify priority areas for further upgrades and enhancements.

### 3.2. Participants

Nursing home size, based on the number of beds in most cases, ranges from small (1–49 beds), to medium (50–99 beds), to large (100 or more beds). Total of 31 participants (18 Male and 13 Female) were recruited in a small nursing home in this research. Our study is classified as quasi-experiment design rather than experimental design. Participants’ features that might affect the phenomena of interest were not randomized or controlled. Quasi-experiment design can minimize threats to ecological validity as natural environments do not suffer the problems of artificiality as compared to an experimental setting. In other words, it maximizes internal and external validity. Recruitment occurred over a 3-month time period in one nursing home in Taoyuan, Taiwan. All participants had resided in nursing homes for more than 18 months and thoroughly understood the services provided by the nursing home. As shown in Table 3, a total of 31 elderly were selected and males (74.2%) outnumbered females (25.8%). Their average age was 82.3 years old, and most of them were above 80 years old (38.7%). The average length of stay in a nursing home was 8.5 years, with most stays falling 6–10 years (41.9%). The participants were asked for their responses based on the service attributes in the questionnaire, and interviewers assisted them with the answers. The process of interviewing took around 45 min for each participant.

### 3.3. Procedure

According to the SERVQUAL instrument proposed by Parasuraman et al. service quality is the degree and direction of discrepancy between customers’ perception of what they received and their expectations of the service [18,19,20]. Only the customers can truly define service quality and the relative importance of attributes. First, each participant received a questionnaire that described all five of the SERVQUAL dimensions. Then they were instructed to consider how important each of the five dimensions was to them and, based on their considerations, to allocate a total of 100 points among the five dimensions. Finally, the participants evaluated their expectations of the service provided by the nursing home and the degree to which they perceived those expectations were met. To identify participants’ expectations, one item asked, “How is your expectations about these service attributes?”, which was scored on a 5-point Likert-type scale of “not at all expected (1)” to “very expected (5).” To identify participants’ perceptions, one item asked, “How do you feel about these service attributes that are already provided?”, which was scored on a 5-point Likert-type scale of “very bad (1)” to “very good (5).” The question for identifying the importance of attributes from participants’ perspective was, “How important are these service attributes?”, scored on a 5-point Likert-type scale ranging from “not at all important (1)” to “very important (5)”. Many researches of SERVQUAL ask participants to complete all the questionnaires in one interview. Take elderly’s response into account, we conducted two to three interviews with each participant to complete all the questionnaires.

### 3.4. Data Collection and Analysis

This method is descriptive statistics rather than inferential statistics. In other words, there is not any assumption for the sample size. The result drawn from multiple nursing homes has better external validity. On the other hand, the recommendations drawn from single nursing home is specific to itself. The differences between participants’ scores on perception (P) and expectation (E) were calculated to determine the gap scores for each service attribute, each of which was calculated separately. For each attribute, the SERVQUAL score (SS) was calculated as the perception score (P) minus the expectation score (E), as illustrated in Equation (1):

SERVQUAL score (WS) = P − E(1)

P: the individual’s Perceptions of given service deliver; E: the individual’s expectations of a given service delivery. The average score of each dimension was calculated to obtain the weighting factor (WF). Then the original SERVQUAL score for each attribute was multiplied by its dimension weighting factor to obtain the weighted SERVQUAL score (WSS), using Equation (2) [31]:

Weighted SERVQUAL score (WSS) = WF × SS(2)

WF: weighting scores, points divided among five dimensions; SS: SERVQUAL score on each attribute.

## 4. Results

### 4.1. Results of Perception Questionnaires Analysis

The mean score of the perception questionnaire was 3.62, indicating that the participants rated the service performance as a little higher than medium (shown as Table 4). The top three items of the perception questionnaire were RL6: “All elderly activities are well scheduled” (mean = 4.00), T9: “The scent in every room is refreshing” (mean = 3.94), and E1: “Employees are helpful, careful, and friendly” (mean = 3.89), respectively.

### 4.2. Results of Expectation Questionnaires Analysis

The mean score of the expectation questionnaire was 4.40, indicating that the participants had high expectations toward the service attributes. The top three items of the expectation questionnaire were RL5: “The employees solve the elderly’s problems sincerely” (mean = 4.68), T3: “Clean, adequate supplies, and well-maintained rooms” (mean = 4.68) and T9: “The scent in every room is refreshing” (mean = 4.65), respectively.

### 4.3. Results of SERVQUAL Questionnaires Analysis

Comparison of the mean scores of expectations and performance questionnaire, the results clearly show a negative service gap in all dimensions (shown as Table 4). The negative values indicated that the nursing homes were not meeting the expectations of their customers. Nineteen attributes were observed as significant differences with *p* < 0.01. The top three gaps between expectations and perceptions existed in T5: “Suitable temperature in the rooms of the elderly” (gap = −1.20), A1: “Feel safe and feel at home” (gap = −1.11) and RL2: “Medical treatments are well explained” (gap = −1.02). However, raw SERVQUAL scores do not consider the relative importance of the service dimensions, so conclusions should not be drawn directly from them. To compensate, the weighted scores were calculated, and the results are presented as follows.

In terms of SERVQUAL dimensions, the nursing homes were performing quite well in the tangible and reliability dimensions. For the tangible dimension, five of the nine attributes were evaluated as high satisfaction (located in Quadrants I and IV). For the reliability dimension, three of the six attributes were evaluated as high satisfaction (located in Quadrants I and IV), and the remaining three attributes were evaluated as low satisfaction with high importance (located in Quadrant II). For the responsiveness, assurance, and empathy dimensions, most of the attributes were in Quadrants II and III and evaluated as low satisfaction.

### 4.4. Results of Important Performance Analysis (IPA)

**Figure 5:**
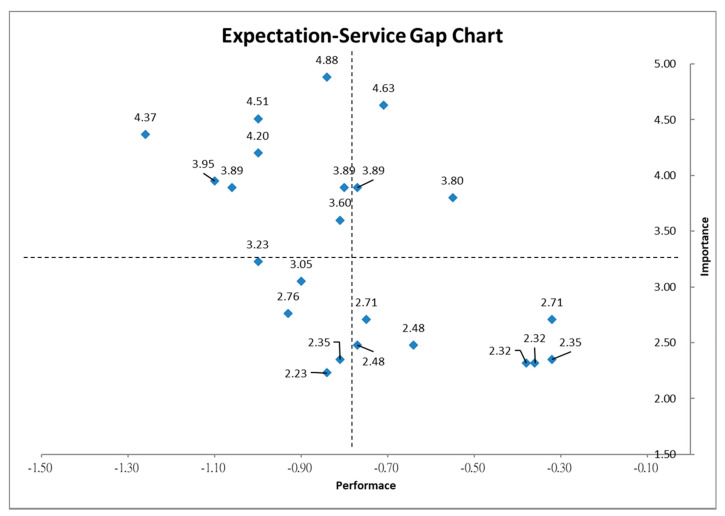
Importance-performance grid.

The importance-performance relationship was transformed into a grid for identification of attributes that had severe service gaps. The SERVQUAL gaps for the 23 attributes were plotted against their importance scores, where importance values form the vertical axis, while performance values form the horizontal axis. This grid presented a macro view of the quality of service delivered by the nursing home (Figure 5). The literature on the use of IPA indicates that the selection of the crosshairs should consider management’s goals for the study in question, and if possible, should force at least one attribute into each of the four quadrants [32].

The IPA grid was divided into four quadrants using the mean expectation score (3.33) and mean gap score (−0.78). Each quadrant in Figure 4 was defined by the level of importance and service gap. It can be seen that Quadrant I had 3 items (T9, E1, R1). These attributes were evaluated as low service gaps but with high importance. This evaluation implies that, for these attributes located in Quadrant I, the current practice should be continued. Quadrant II had 8 items (T3, T5, RL1, RL2, RL5, R2, E2, E3). These attributes were evaluated as high service gaps with high relative importance. These evaluations imply that these attributes in Quadrant II should be improved and more resources should be allocated. Quadrant III had 5 items (T6, T8, R3, A1, A2). These attributes were evaluated as low service gaps with high relative importance. These results imply that the attributes in this quadrant could be improved but not prioritized. Quadrant IV had 7 items (T1, T2, T4, T7, RL3, RL4, RL6). These attributes were evaluated as low service gaps with low importance, implying that these attributes may be unnecessary. The items in Quadrants II and III require corrective actions, as they have high service gaps with high and low importance, respectively. Conversely, items in Quadrants I and IV can be accorded lower priority, as the satisfaction was at an acceptable level.

## 5. Discussion

The overall dissatisfaction of the residents was comprehensively considered in the SERVQUAL gap scores, IPA and statistical significance. The focus of the recommendations was on addressing Quadrant II of the IPA matrix, which had significant differences (*p* < 0.01) between residents’ expectations and perceptions. Attributes under these criteria were considered important by the residents, but they did not think the performance met their requirements. The e-Health system should be adopted to improve the service quality of nursing homes. The critical dissatisfactions of the elderly residents can be classified into three factors—environment, medical treatment, and staff service—and three dimensions: physical environment quality (consisting of ambient conditions, design, and facilities), interaction quality (consisting of attitude, behavior, and experience), and outcome quality (consisting of waiting time, tangibles and value) [33]. The quality of service is not an operational issue alone, for the psychological side of the residents should be considered.

Regarding the physical environment quality, the residents did not think the nursing homes provided clean, well-maintained rooms with adequate supplies (T3). In addition, the temperature in their rooms was not suitable (T5). Our in-depth interviews of the residents revealed that their needs for the living environment were varied. Even though residents could set the room temperature and call for service themselves with a service button, they always complained about the room temperature and cleanliness. A likely explanation is that the residents did not make good use of the thermostats and service button to adjust the temperature and keep the environment at their desired level of cleanliness. Elderly residents did not make good use of technology because the system designers, assuming that they had similarities with average potential users, failed to consider age-related differences (e.g., reduced working memory capacity, motor learning and performance, attention control, vision and hearing). Therefore, the mental models of the elderly on how technology works might be not supported when modern technologies become increasingly complex. Elderly residents may tend to be hesitant and have negative attitudes toward learning how to use unfamiliar technological devices; such attitudes present a barrier and limit the effectiveness of the e-Health system. 

Our suggestion for reducing this gap is to understand the characteristics of elderly residents and the ways aging shapes their perceptions and behaviors in using such a system. These characteristics need to be incorporated into the design process as crucial factors of the technologies that the elderly want. On the other hand, technology training courses suitable for elderly residents need to be arranged, and the residents need to be encouraged to participate. Efficient learning could produce a sense of accomplishment. This positive feeling could promote their self-esteem regarding interactions with technological products.

Regarding the outcome quality, the elderly residents did not think the medical treatments and doctor visits were well scheduled (RL2). Our in-depth interviews of the residents revealed that they spent much time waiting for rehabilitation services. Furthermore, scheduled doctor visits were often delayed by unexpected matters, leading to long waits for medical care. Punctuality and regularity of medical services would make the elderly feel at ease and secure. Furthermore, they should be notified in advance of any changes to their medication or health care. A sense of participation would likely make the elderly residents feel highly respected and have positive self-esteem in the organization. In addition, predictability could stabilize the elderly residents’ emotions efficiently, making them feel at home. ICT tools can provide remote interaction between nursing home residents and health professionals. For example, health care informatics (HCI) are software solutions for appointment scheduling, patient data management, work schedule management and other administrative tasks surrounding health. These solutions automatically provide real-time information and reminders to residents. Thus, any changes in the scheduled medical treatments and doctor visits will be known to the residents. Cases of adopting ICT to reduce waiting time and improve scheduled medical services successfully have been demonstrated in previous research [34].

Regarding the interaction quality, the residents did not think the staff solved their problems sincerely or without discrimination (RL5, E3). Furthermore, the residents indicated that the staff did not understand their needs exactly or provide appropriate and prompt services (RLI, R2). Consistent with previous research on nursing homes, this study found that it is more important for nursing staff to have a caring attitude than to have a high level of training [35,36]. The importance of communication between nursing staff and the residents in nursing homes has also been explored by Lee and Liu [37]. Their research showed that delivering a clear message about service features and the services they provide is an efficient way to improve the residents’ satisfaction in long-term care service stations. The difference between healthcare services and other services is that trust must be established between the medical staff and residents [38,39]. Proper responses to residents’ requests are crucial because a professional, friendly, and enthusiastic attitude can make residents feel the staff are reliable and increase the residents’ sense of security. These service gaps may be related to the medical staff’s training, experience, empathy, and understanding of the residents’ psychological needs [40]. The skills for interacting with the elderly could be improved by on-the-job training and experience sharing to increase the competency levels.

Information technology has a tremendous impact on interaction quality, especially on the interactions between nurses and residents in nursing homes. First, nurses and medical staff cannot provide optimum service if their knowledge and information levels are insufficient [4]. They could obtain that information from electronic health records (EHR), which are digital records of health information containing all the information normally on paper charts. These records include medical history, vital signs, progress notes, diagnoses, medications, immunization dates, allergies, imaging reports, and even insurance information and demographic information [41]. The power of EHR lies not only in the information they contain, but in how that information is shared. EHR make health information instantly accessible to authorized staff, providing updated information on residents’ conditions and facilitating the coordination of care. With the help of EHR, medical staff can understand residents’ needs exactly and provide appropriate and prompt services.

Interviews of the residents also revealed that service gaps were much more likely to occur due to nursing shift changes. In healthcare, a shift report is a meeting between healthcare providers at the change of shift in which vital information about and responsibility for the residents is passed from the off-going provider to the on-coming provider [42]. An electronic shift system (ESS) can update residents’ information via links to the patient database and reduce the time required for shift reports by enhancing the efficiency of information management. In addition, an ESS can generate alerts or reminders about changes in resident status. Such a system is very helpful for the medical staff in making decisions when directing care plans and delivery for nursing home residents. Previous studies have found that introducing an ESS leads to improved accuracy and completeness of documentation in a way that it is logically organized and easily retrieved [43].

## 6. Conclusions

The various forms of e-Health systems are progressively showing their advantages over traditional healthcare, but acceptance of such systems is still a challenging issue. Evaluation is needed to uncover the actual performance and to determine the next step. The scope of e-Health evaluation can cover the entire life cycle, which spans the planning, design, implementation, use, and maintenance of the e-Health system over time. For each stage being evaluated, the focus can shift. In the pre-implementation stage, managers need information about dissatisfaction with the service quality and the organization’s overall culture to prioritize the implementation. According to previous research, an estimated 80% of technology projects fail because of a lack of organizational willingness to change [44]. In addition, recent studies have suggested that nursing homes have had limited preparation for e-Health system implementation because leadership tends to have a poor understanding of ICT. Furthermore, users’ comments should be addressed in the planning phase, prior to e-Health system implementation. What this indicates is that managers have a high degree of uncertainty because of their lack of sufficient information on how to adopt ICT and comments collected from users. Scholars have called for further research on the adaptation of e-Health technology in medical organizations.

In response to that call, an instrument to evaluate e-Health service quality in nursing homes was developed based on the SERVQUAL model. Furthermore, a pre-implementation analysis combining a SERVQUAL questionnaire and importance performance analysis was performed. Service quality shortfalls in nursing homes were identified, and the corresponding ICT applications and systems were recommended as solutions. Research has shown that the quality of care in nursing homes has continued to decline as funding and staffing resources have plunged in recent years. Under limited resources, our instrument and methodology proposed in this research could contribute to the efforts to stop that decline.

## 7. Limitations

The results obtained in this research have several limitations. First, the gender distribution was not broadly symmetrical, and the participants were generally male (74.4% males vs. 25.8% females). Another limitation of the study is that it was performed in single nursing homes in northern Taiwan, and aging properties differ among various regions and socio-cultural groups. It is dubious whether recommendations drawn up in this research could be generalized to other nursing homes. Furthermore, without the criterion-related validity from third party, the criticism of pigeonholing may have occurred. The recommendations drawn from this research should be regarded as a case study. The main purpose in this study is to propose a validated and reliable SERVQUAL questionnaire specific to nursing homes. This questionnaire can reveal multidimensional service defects, and is especially suitable for the pre-adoption evaluation of novel technology for nursing homes. Our following researches will focus on the performance of medical staff and the comparison of residents’ satisfaction before and after the adoption of ICT technologies.

## Figures and Tables

**Table 1:** Five SERVQUAL dimensions and descriptions applied in this study.

Dimensions	Descriptions	Example of Nursing Homes
Tangible	The appearance of physical facilities, equipment, personnel, and communication materials.	The physical facilities of health services institution would be visually attractive, e.g., buildings, medical equipment, and the appearance of staff etc.
Reliability	The ability to perform the promised service dependably and accurately.	Services are provide at the time they promise to do so, e.g., medical investigations, treatment, food etc.
Responsiveness	The willingness to help customers and to provide prompt service.	Staff shows a sincere interest in solving problems and willing to help elderly.
Assurance	The knowledge and courtesy of employees and their ability to convey trust and confidence.	Staff has the knowledge to answer questions and acts courteous with elderly.
Empathy	The provision of caring, individualized attention to customers.	Staff has the patient’s best interest at heart and build with them long-term relationships

**Table 2:** The SERVQUAL attributes applied in this study.

Dimension	NO	Code	Service Attributes
Tangible	1	T1	Medical instruments and physical facilities are visually appealing.
	2	T2	Employees’ uniforms are clean, nice, and neat.
	3	T3	Clean, adequate supplies, and well-maintained rooms
	4	T4	Good lighting in every room
	5	T5	Suitable temperature in the rooms of the elderly
	6	T6	Meals served are clean and hygienic.
	7	T7	Meals served are delicious.
	8	T8	The atmosphere of every room is cozy.
	9	T9	The scent in every room is refreshing.
Reliability	10	RL1	Appropriate employee responses
	11	RL2	Medical treatments are well explained.
	12	RL3	Available and adequate family visiting times
	13	RL4	All elderly activities are well scheduled.
	14	RL5	The employees solve the elderly’s problems sincerely.
	15	RL6	All equipment (AC, TV, radio, lights, etc.,) works properly.
Responsiveness	16	R1	Employees give clear, understandable information
	17	R2	Appropriate and prompt services
	18	R3	Quick medical treatment response when the elderly need it
Assurance	19	A1	Feel safe and feel at home
	20	A2	Employee behavior instills confidence in the elderly.
Empathy	21	E1	Employees are helpful, careful, and friendly.
	22	E2	Nurses understand the elderly’s needs.
	23	E3	No discrimination against the elderly

**Table 3:** Participants’ demographics.

Demographics	Categories	Frequency	Percentage
Gender	Male	23	74.2%
	Female	8	25.8%
	51–60	3	9.7%
	61–70	9	29.0%
	71–80	7	22.6%
	>80	12	38.7%
Length of stay (years)	1.5–5	6	19.4%
	6–10	13	41.9%
	11–15	7	22.6%
	>20	5	16.1%

**Table 4:** The results of SERVQUAL analysis.

Dimension	Code	Performance		Expectation		Gap	Importance Scores	Pair *t*-Test	
		Mean	SD	Mean	SD			*t*-Value	*p*-Value
Tangible	T1	3.87	1.29	4.19	1.38	−0.32	2.35	−0.9	0.19
	T2	3.81	1.18	4.19	1.15	−0.38	2.32	−1.20	0.12
	T3	3.84	1.08	4.68	0.69	−0.84	4.88	−4.82	<0.01 **
	T4	3.65	1.15	4.29	1.08	−0.64	2.48	−2.65	0.01 **
	T5	3.35	1.23	4.61	0.66	−1.26	4.37	−5.22	<0.01 **
	T6	3.39	1.29	4.23	1.10	−0.84	2.23	−2.79	<0.01 **
	T7	3.48	1.36	4.23	1.10	−0.75	2.71	−2.39	0.01 **
	T8	3.52	0.95	4.42	0.91	−0.90	3.05	−4.32	<0.01 **
	T9	3.94	0.95	4.65	0.70	−0.71	4.63	−3.32	<0.01 **
Reliability	RL1	3.61	1.10	4.42	1.19	−0.81	3.60	−3.76	<0.01 **
	RL2	3.48	1.29	4.58	0.75	−1.10	3.95	−5.85	<0.01 **
	RL3	3.52	1.34	4.29	1.37	−0.77	2.48	−2.68	0.01 **
	RL4	3.77	1.13	4.13	1.36	−0.36	2.32	−1.19	0.12
	RL5	3.68	1.09	4.68	0.59	−1.00	4.51	−4.95	<0.01 **
	RL6	4.00	0.92	4.32	1.17	−0.32	2.71	−1.19	0.12
Responsiveness	R1	3.68	1.09	4.45	1.04	−0.77	3.89	−4.08	<0.01 **
	R2	3.39	1.01	4.45	1.10	−1.06	3.89	−4.79	<0.01 **
	R3	3.39	0.97	4.32	1.20	−0.93	2.76	−4.21	<0.01 **
Assurance	A1	3.19	1.18	4.19	1.28	−1.00	3.23	−4.23	<0.01 **
	A2	3.58	1.19	4.39	1.07	−0.81	2.35	−3.32	<0.01 **
Empathy	E1	3.90	0.96	4.45	0.94	−0.55	3.80	−2.53	0.01 **
	E2	3.58	1.16	4.58	0.79	−1.00	4.20	−4.08	<0.01 **
	E3	3.68	1.12	4.48	0.98	−0.80	3.89	−3.59	<0.01 **

Note: * *p*-value is less than 0.05; ** *p*-value is less than 0.01.
